# Empirical Driven Automatic Detection of Lobulation Imaging Signs in Lung CT

**DOI:** 10.1155/2017/3842659

**Published:** 2017-03-29

**Authors:** Guanghui Han, Xiabi Liu, Nouman Q. Soomro, Jia Sun, Yanfeng Zhao, Xinming Zhao, Chunwu Zhou

**Affiliations:** ^1^Beijing Key Laboratory of Intelligent Information Technology, School of Computer Science and Technology, Beijing Institute of Technology, Beijing, China; ^2^Department of Software Engineering, Mehran University of Engineering and Technology, SZAB Campus, Khairpur Mir's, Pakistan; ^3^Department of Imaging Diagnosis, Cancer Institute and Hospital, Chinese Academy of Medical Sciences, Beijing, China

## Abstract

Computer-aided detection (CAD) of lobulation can help radiologists to diagnose/detect lung diseases easily and accurately. Compared to CAD of nodule and other lung lesions, CAD of lobulation remained an unexplored problem due to very complex and varying nature of lobulation. Thus, many state-of-the-art methods could not detect successfully. Hence, we revisited classical methods with the capability of extracting undulated characteristics and designed a sliding window based framework for lobulation detection in this paper. Under the designed framework, we investigated three categories of lobulation classification algorithms: template matching, feature based classifier, and bending energy. The resultant detection algorithms were evaluated through experiments on LISS database. The experimental results show that the algorithm based on combination of global context feature and BOF encoding has best overall performance, resulting in *F*1 score of 0.1009. Furthermore, bending energy method is shown to be appropriate for reducing false positives. We performed bending energy method following the LIOP-LBP mixture feature, the average positive detection per image was reduced from 30 to 22, and *F*1 score increased to 0.0643 from 0.0599. To the best of our knowledge this is the first kind of work for direct lobulation detection and first application of bending energy to any kind of lobulation work.

## 1. Introduction

Lung disease is one of the fatal diseases for human. Statistics of the World Health Organization (WHO) showed 1.59 million deaths occurred alone in 2012 due to the lung cancer [1]. The fatality ratio of lung cancer can be minimized if the cancer sings are detected and treated earlier. Here, the computed tomography (CT) examinations play an important role in early detection and classification of lung lesions.

Lung nodule is currently a most concerning type of lung lesions. We regard what radiologists see in lung nodules for diagnosing diseases as CT imaging signs, which are also often called “CT features,” “CT findings,” “CT patterns,” or “CT manifestation.” These CT imaging signs are very crucial in disease diagnosis and research works [2].

**Figure 1:**
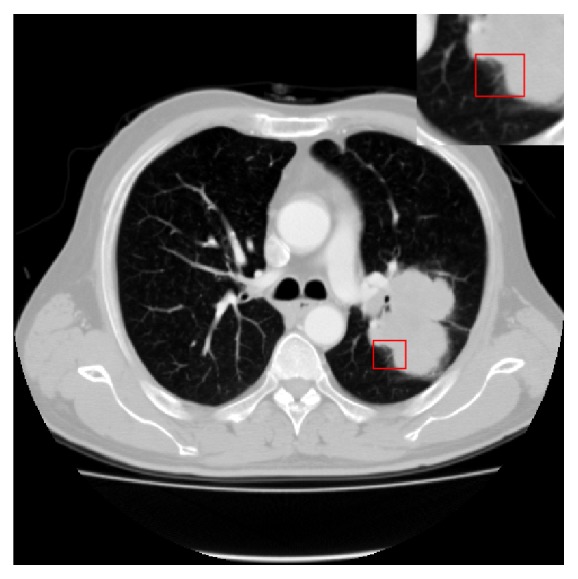
An example of annotated lobulation CT image, where the red rectangle indicates the lobulation region annotated by the radiologist.

*Lobulation* is an important category of CT imaging signs, which is dependent on the ingrowth of connective tissue septa containing fibroblasts derived from perithymic mesenchyme [3]. It is normally related with malignant lesion, though it also occurs in up to 25% of benign nodules [4, 5]. Visually, a lobulation shows the indentation which appears at the edge of round or oval lesion.  Figure 1 shows an example of lobulation, where the small rectangle indicates the region annotated by the radiologist and is magnified to be displayed more clearly in the bigger rectangles overlapping in the image.

The slice number of CT scan generated in a CT examination has become larger and larger in recent years, so it is tedious and laborious for the radiologist to read CT image slice by slice. The Computer-aided detection (CAD) technique has the potential to increase working effectiveness and efficiency of radiologists. Though there were some research works related to automatic detection of lesions [6, 7], automatic detection of lobulation signs has not been addressed properly, except some work related to classification of lobulation signs. Kovalerchuk et al. [8] proposed an approach for discriminating lobulation from microlobulation and showed that the fuzzy logic is an effective tool in dealing with this problem. The authors argued that their approach is designed in a manner which copies the way human experts make decisions. Iwano et al. [9] found that the mean circularity of nodules has the following hierarchy (from large to small): round, polygonal, lobulated, speculated, tentacular, ragged, and irregular. Nevertheless, lobulation and polygonal signs showed little difference in their formation. By contrast, the mean second moment was entirely different. They believed that the combination of circularity and second moment can easily differentiate polygonal from lobulated nodules and vice versa. In the solution, they used another image processing software package to extract and analyze pulmonary nodules (the authors did not give more information about the software package). Ekarin Varutbangkul and Furst [10] proposed a framework for pulmonary nodule interpretation in thoracic CT images. They used logistic regression to predict categories, including lobulation nodule. The generalized logits model and cumulative logits model were applied for nominal and ordinal characteristics, respectively. For every model, a response probability is obtained. Then the highest response probability is used for deciding the predicted category.

To the best of our knowledge, automatic detection of lobulation imaging signs has not attracted enough work in the literature thus far. By contrast, the existing classification methods took the segmentation results of nodules or masses as its input. But lesion segmentation itself is difficult and unsolved well yet. In this paper, we designed a sliding window based framework for lobulation detection. Under the proposed framework, several algorithms with different classification techniques were investigated. In this paper, we contributed following:

- In contrast to existing lobulation classification methods, sliding window is used to detect the lobulation directly without preprocessing of nodule or mass.
- To the best of our knowledge, this is the first work for direct automatic lobulation detection and bending energy is first time used for lobulation signs detection.
- Empirical driven local features, template matching, encoding methods, and their best combinations are suggested to improve precision and reduce false positive.

The rest of this paper is organized as follows.  Section 2 describes the sliding window based algorithm framework for lobulation detection and three categories of corresponding algorithms. In Section 3, we evaluate the performance of these solutions experimentally. In Section 4, the challenges of lobulation detection and the possible strategies to further improve the detection effects are discussed. We conclude in Section 5.

## 2. The Proposed Lobulation Detection Algorithms

As mentioned in Section 1, automatic lesion segmentation is difficult and unsolved well yet. In order to avoid negative impact from wrong segmenting of nodules or masses, we design a sliding window based framework for lobulation detection in this section. The sliding window method has been used in many research works [11–14]. By adopting traditionally sliding window based method, we achieved many advantages: (i) Get rid of need of segmented nodules drawn by radiologists or software packages as preprocessing step hence paving the way for direct automatic detection. (ii) Eliminate the probability of misdetection of nodule which directly affects the lobulation classification. 

**Figure 2:**
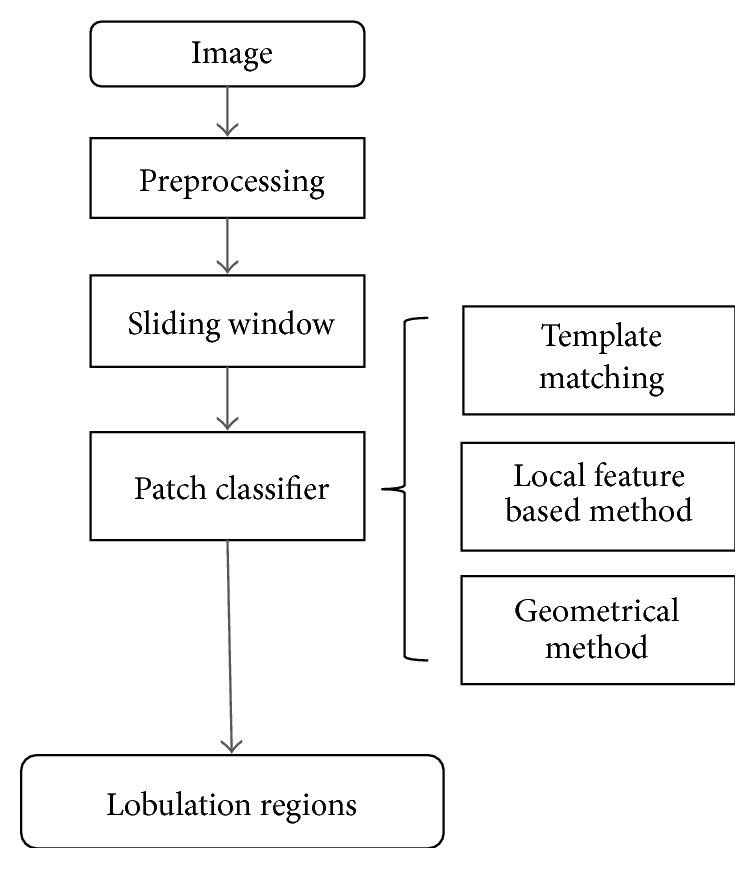
The flowchart of our proposed detection framework.

The flowchart of our detection framework is illustrated in Figure 2. As shown there, the inputted image is transformed in HU unit and lung parenchyma is segmented roughly. Then the sliding window method is used on the lung parenchyma to obtain local patches in sequence. Finally, the classifiers are used to classify each patch into lobulation or not. In this paper, three categories of classifiers, including template matching, local feature based classifiers, and geometrical method, are considered and compared with each other.

### 2.1. Preprocessing

**Figure 3:**
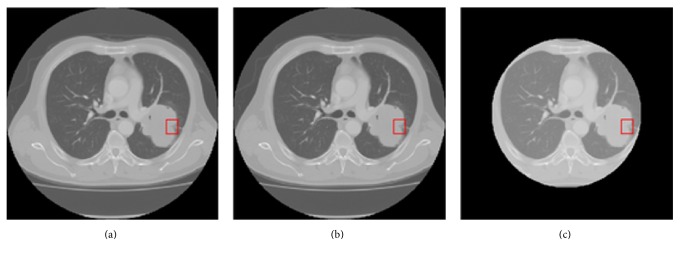
Image preprocessing: (a) the original image, (b) the rescaled image, (c) the segmented lung parenchyma. The red box is lobulation region annotated by radiologist.

The preprocessing of CT images in this paper includes the following parts. Firstly, the gray value in CT image is converted to CT value (in Hounsfield Unit, HU). Then lung parenchyma is segmented to reduce the disturbance of other regions.  Figure 3 illustrates the preprocessing steps. The details of each step are introduced as follows.

#### 2.1.1. CT Value Determination

**Figure 4:**
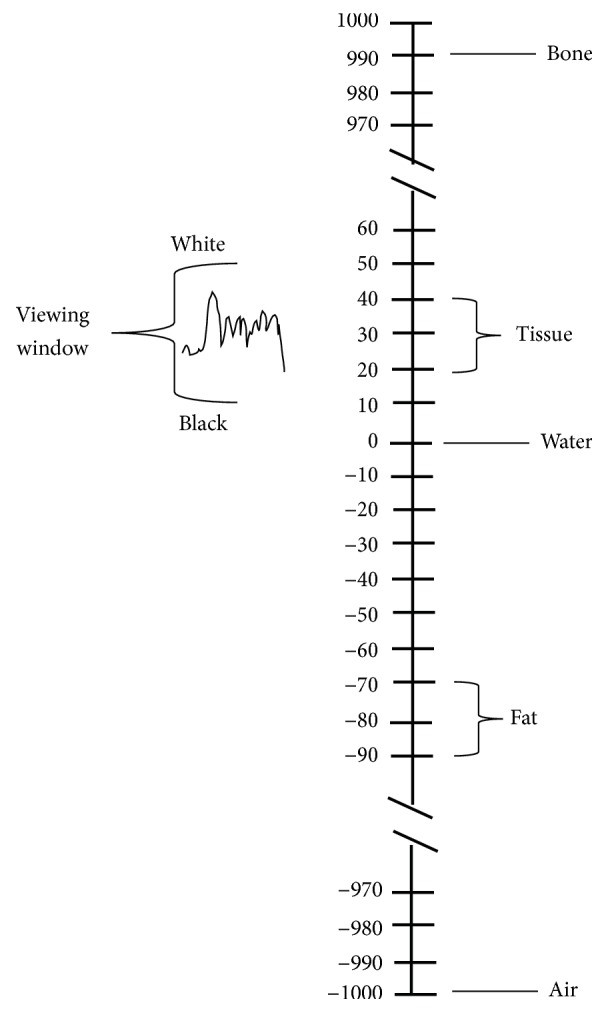
Demonstrating the accuracy to which absorption values can be ascertained on the CT picture.

Figure 4 demonstrates the accuracy to which the absorption values can be ascertained on the picture, in which absorption value of water was chosen to be zero at the center for CT imaging. The whole range, from air (−1000) at the bottom of the scale to the bone at the top of the scale, covers some 1000 levels of absorption of either side of the water. To obtain readings which relate to true absorption, 1000 must be added to these readings, making air zero, and water would then be +1000 approximately [15]. Therefore, the normal units found in CT data (a typical data set ranges from 0 to 4000 or so) should be rescaled to obtain the data in HU units. This transformation can be done by using the formula

(1) $$\begin{array}{c} HU\left(\;x_{0},y_{0}\;\right)=K\times f_{xy}\left(\;x_{0},y_{0}\;\right)+C, \end{array}$$

where HU denotes CT value and *f*(*x*_0_, *y*_0_) is the gray value of pixel at row *x*_0_ and column *y*_0_ in CT image. The parameters *K* and *C* are rescaled slope and rescaled intercept, respectively, which can be obtained from the metadata of DICOM file.

#### 2.1.2. Lung Parenchyma Segmentation

**Figure 5:**
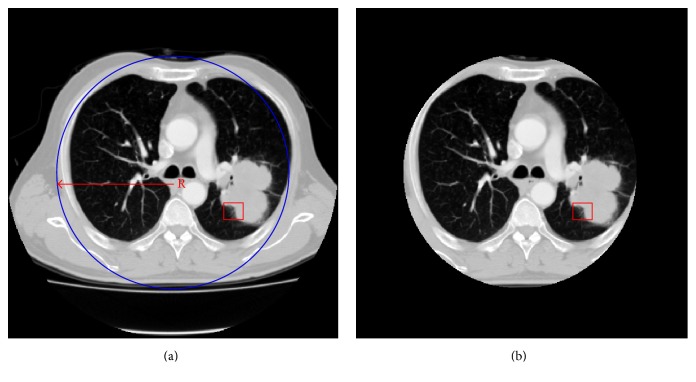
Lung parenchyma segmentation: (a) estimate the maximum radius *R*; (b) segment the circle region with radius *R* as lung parenchyma. The red rectangle indicates the lobulation region annotated by the radiologist.

We segment the lung parenchyma in CT image in two steps. First, we estimate the maximum radius *R* of lung parenchyma roughly through lung CT images, which is suitable for all images. Because CT scanning is performed at a preset position of human body, the lung parenchyma is always located near the image center. In our experiments, we take the image center as the center of considered circle which has the ability to cover lung parenchyma. The maximum radius *R* is set to be 180 pixels in this paper through the process of estimating the maximum radius of lung parenchyma. Second, we segment the lung parenchyma according to the maximum radius *R*. The segmentation result is rough because the shape of lung parenchyma is not circle exactly. But still, processing of segmentation result is much better than processing of whole image in the next stage.  Figure 5 shows an example of such lung parenchyma segmentation.

### 2.2. Template Matching

To explore and evaluate the performance of model based methods for lobulation, template matching is considered. In template matching based classification method, the template acquisition method is important. In practice, multiple different templates are usually created in order to adapt to different orientations and length-width ratios of actual objects. Then the similarity between the template and object region is computed for determining whether the object region is labeled as the matched region.

To deal with the lobulation detection problem in this paper, we select eight typical sample Regions of Interests (ROIs) for eight different orientations, which almost illustrate all the types of lobulation signs. There is an approximately 45-degree interval between the two adjacent orientations. For each orientation, the selected sample ROI is transformed with 6 length-width ratios and 3 scaling factors, respectively, so total 72 templates are finally obtained. All these templates are used to match the lobulation sign in the target image. Selected ROIs are from the LISS database [2], which are annotated by radiologists.

**Figure 6:**
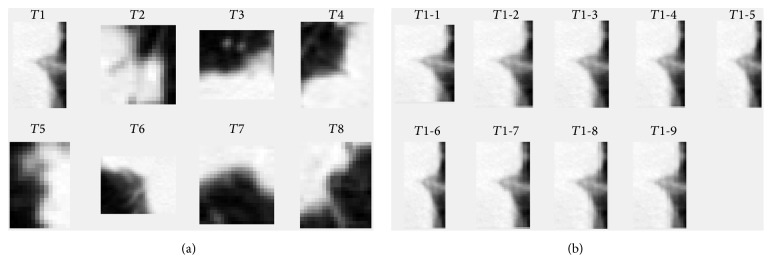
(a) Templates from eight different orientations. (b) Template *T*1 is transformed with 6 length-width ratios and 3 scaling factors, respectively.

Figure 6 illustrates the transformation of image proportion. The length-width ratio in Figure 6 denotes the ratio between the side lengths of image before and after the transformation. For example, the ratio 1 : 0.8 means that the length remains unchanged, and width is transformed into 0.8 times of initial value. We consider six length-width ratios: 1 : 0.8, 1 : 0.9, 1 : 1 (original), 1 : 1.1, 1 : 1.2, and 1 : 1.3, respectively. Furthermore, the template is transformed by 3 scaling factors, respectively: 1.1, 1.2, and 1.3. Therefore, take just the example of template* T*1: 9 variant templates, *T*1-1, *T*1-2,…, *T*1-9, are obtained, as illustrated in Figure 6.

We took the standard normalized cross correlation (NCC) value between a template and an image patch as the matching degree between them. Let *f*(*x*) be the template, *g*(*x*) be the image patch, and then the standard NCC can be calculated by using

(2) $$\begin{array}{c} NCC=\frac{\sum f\left(x\right)\times g\left(x\right)}{\left|f\left(x\right)\right|\times \left|g\left(x\right)\right|}. \end{array}$$

The larger the value is, the higher possibility they match. It is independent of illumination and only dependent on texture.

**Figure 7:**
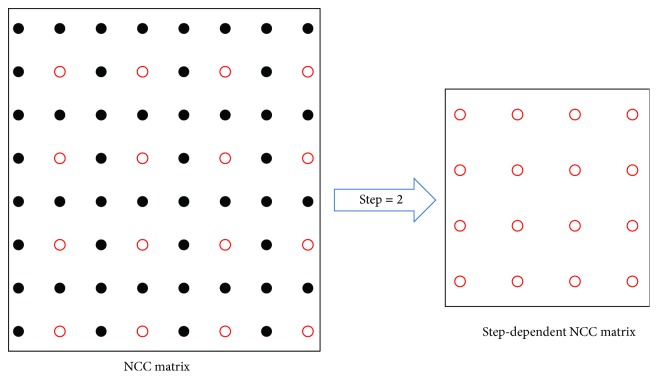
Step-dependent NCC matrix.

Our algorithm of detecting lobulation signs based on template matching is described in Algorithm 1. To accelerate the computing speed, the standard NCC value of each pixel in target image is computed with respect to frequency domain; then a NCC matrix is computed. Each element of NCC matrix is the NCC value between the template and the corresponding region (with same central location). Then we select corresponding locations of NCC matrix according to sliding step to form new step-dependent NCC matrix as shown in Figure 7. After the step-dependent NCC matrix is constructed for a template, each step-dependent interesting point has a response NCC value. Then the highest response NCC value in the matrix is used for deciding the matched location candidate and its location (*x*_*i*_, *y*_*i*_) is recorded. Finally, the algorithm outputs the top *N* regions (for all templates) sorted by the NCC value related to it.

### 2.3. Local Feature Based Classifiers

Local image features are widely used for object detection and image classification tasks in recent years. Researchers have also proposed several encoding methods to represent images based on local image features. However, lobulation imaging sign is hard to define with its intrinsic undulated nature in size, different number, no uniformity, and existence of both deepness and shallowness. So many contemporary methods failed to extract all features of lobulation imaging signs and make this research area stagnant. Keeping this in view, we revisited literature and design several best lobulation detection algorithms based on local image features and encoding methods. The image features considered in this paper include PHOW (also known as dense SIFT) [16], HOG [17], shape context [18], global context [19], and LIOP [20]. The considered encoding methods include the Bag of Features (BOF), Fisher Vector (FV), and Vector of Locally Aggregated Descriptors (VLAD). In our detection framework, the local features are encoded by the encoding method and then are inputted into the SVM classifier for classification.

#### 2.3.1. Image Features

1. *PHOW*. The Pyramid Histogram Of visual Words (PHOW) feature is simple dense Scale Invariant Feature Transform (SIFT) applied at several resolutions, which are formed by using the appearance together with the image spatial layout [21]. The PHOW feature of an image is a 128 × *N* matrix, where 128 is the feature dimension and *N* is the number of key points extracted from the image. The local descriptors based on SIFT are computationally efficient and have been proven highly effective features for many applications.
2. *HOG*. HOG feature [17] evaluates well-normalized local histograms of image gradient orientations in a dense grid. For an object, the shape and local appearance can be defined well by the distribution of edge directions or local intensity gradients. For the lobulation sign, the gradient orientations are obvious, so we consider the HOG feature in this paper.
3. *Shape Context*. Belongie et al. [19, 22] proposed the Shape Context (SC), which is a scale and rotation invariant local descriptor attached to each point. The shape context at a reference point captures the distribution of the remaining points relative to it, thus offering a globally discriminative characterization [22].
4. *Global Context*. The global context (GC), similar to shape context, is helpful to discriminate local features that have a similar local appearance. However, the global context computes the maximum curvature at each pixel [19], instead of counting the distinct edge points, because they can be sensitive to variations in contrast and thresholding values. Like SIFT, global context and shape context also construct a histogram, but here, sampled edge points in each bin of a log-polar histogram are counted over a large portion of the image.
5. *LIOP*. Local Intensity Order Pattern (LIOP) is proposed to encode the local ordinal information of each pixel based on intensity order. The basic principle of LIOP is that the relative order of pixel intensities remains unchanged when the intensity changes are monotonic. Initially, local patch is divided into subregions based on the overall ordinal information and this is also called ordinal bins. Then, a local intensity order pattern of each point is described on the basis of the connection between the intensities of its neighboring sample points. More specifically, LIOPs of points in each ordinal bin are accumulated and then concatenated together to construct LIOP descriptor [20]. Since the regional division and LIOP computation are all based on the relative relationships of intensities, the LIOP descriptor is inherently invariant to image rotation and monotonic intensity changes.

For all aforementioned features, we used it alone or combined it with another one based on the following consideration. The PHOW feature was used alone to verify the effectiveness of three different encoding methods. Because HOG, shape context, and global context features need key points, they are used together with PHOW, and these features used the consistent key points which are extracted by PHOW method. The LIOP was used alone as well as in combination with LBP, respectively.

For the combination of PHOW and HOG, we obtain the PHOW descriptors and key points, firstly, and then use the key points and the original image to compute the HOG descriptors. The SIFT descriptors and HOG descriptors are combined together at the end. For the combination of PHOW and shape context, a canny edge detector is firstly applied to the CT images because the shape context works on the object edge (e.g., shape). Then the shape context feature of ROI is computed and combined with PHOW feature to form fusion feature. For PHOW and global context feature, we try to combine them to form mixture descriptors and then encode the PHOW-GC fusion descriptors using BOF method. Through comparing the experiment with that of shape context, we have a chance to compare the distinguished shape capacity of these two features in lobulation detection. Considering the structural similarity of lobulation with various orientations or intensity, we also test the LIOP feature alone and with the combination of LBP feature for lobulation detection task, respectively.

#### 2.3.2. Encoding Methods

There are several approaches for modeling the distribution of low-level features extracted from images irrespective of their absolute or relative locations within the image. As described above, we investigate three different encoding methods (BOF, FV, and VLAD) for lobulation detection task. We used the VLFeat open source library to implement these encoding methods [23].

1. *Bag of Features (BOF)*. The BOF method forms a group of local descriptors extracted from images. It uses a codebook with *k* “visual words,” which are usually obtained by *k*-means clustering. For an image, each local descriptor is assigned to the closest centroid. The representation of BOF is constructed by assigning the histogram of the assignment of all image descriptors to visual words. Thus, it constructs a *k*-dimensional vector, and that is finally normalized [24].
2. *Fisher Vector (FV)*. FV is an image representation obtained by pooling local image features and is frequently used as a global image descriptor in visual classification [23]. Sánchez et al. [25] proposed a patch aggregation approach established on the principle of Fisher Kernel (FK). In brief, it is characterizing a sample by its deviation from the generative model. The deviation is measured by computing the gradient of the sample log-likelihood with respect to the model parameters. This leads to a vectorial representation which is called Fisher Vector (FV). The FV representation provides a more general way to define a kernel from a generative process of the data. Moreover, it can be computed from much smaller vocabularies to obtain a lower computational cost.
3. *Vector of Locally Aggregated Descriptors (VLAD)*. Jégou et al. [24] proposed a vector representation of an image which aggregates descriptors based on a locality criterion in feature space. It is also viewed as a simple form of Fisher kernel. In addition, there is a similarity between VLAD and BOF. In the BOF, codebook *C* = {*c*_1_,…, *c*_*k*_} of* k* visual words with* k*-means is firstly learned. Each local descriptor *x* is associated with its nearest visual word  *c*_*i*_ = *NN*(*x*). Contrarily, the concept of the VLAD descriptor is to accumulate, for each visual word  *c*_*i*_, the differences *x* − *c*_*i*_ of the vectors *x* assigned to  *c*_*i*_. And this defines the distribution of the vectors with reference to the center. The VLAD encoding is usually normalized before it is used.

#### 2.3.3. Classification

**Figure 8:**
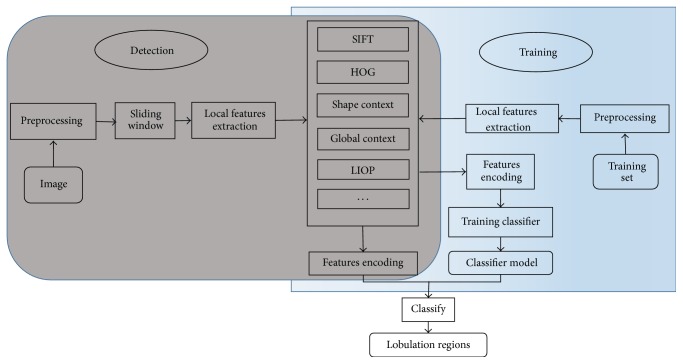
Lobulation detection based on local feature based classifiers.

The local feature based classification procedure is illustrated in Figure 8. For each image patch, the local descriptors are extracted and are encoded by corresponding encoding method which is used in the training stage. Then the encoded features are classified as lobulation sign or normal region by the trained SVM classifier. Based on the image features and encoding methods considered above, we design eight lobulation detection algorithms which are shown in Table 1, in which A*x* denotes Algorithm *x* (similarly hereinafter).

### 2.4. Bending Energy

Bending energy is a curvature-based method for describing the shape of closed contours, in which the curvature is used to describe or measure the bend state of an object. The bending energy of an object denotes the energy stored in its shape. The more sharply one object bends, the higher curvature it has. Young et al. [26] proposed an analysis approach based on the idea of bending energy. In their approach, the bending energy is relying on the total size of the object. The average bending energy per unit length is defined by

(3) $$\begin{array}{c} E=\frac{1}{P}\overset{p}{\underset{0}{\int }}k{\left(\;p\;\right)}^{2}\;dp, \end{array}$$

where *k*(*p*) is the curvature at point *p*, and *P* is the total curve length. The average bending energy per unit length can be computed more efficiently by considering a weighted sum of the Fourier series coefficients for the parametric description (*x*(*p*), *y*(*p*)).

As mentioned in Section 1, a lobulation sign has the indentation at the edge of round or oval lesion. That is to say, the curving characteristic of lobulation contour is essential. In contrast to active contour [27] and level-set [28], which captures the whole object or nodule, bending energy is good for curving characteristics. So it may be a good approach to detect lobulation with bending energy and, to the best of our knowledge, it is for the first time used in lobulation imaging sign. According to this idea, we develop a solution based on the bending energy to reduce false positives of Algorithm 8 and Algorithm 9. Because the bending energy works with the binary image, Otsu's method was used to convert images into binary images. This solution is summarized in Algorithm 10, where *θ*1 and *θ*2 are set to 0.0124 and 0.8, respectively, through careful experiments. In addition, Algorithm 10 takes the output of Algorithm 8 or Algorithm 9 as its input, and we mark Algorithm 10, to A10a (corresponding to Algorithm 8) and A10b (corresponding to Algorithm 9) in order to distinguish between two such different cases.

## 3. Experiments

In this section, we evaluate the above lobulation detection algorithms and present experimental results.

### 3.1. Experimental Setup

#### 3.1.1. Dataset

**Figure 9:**
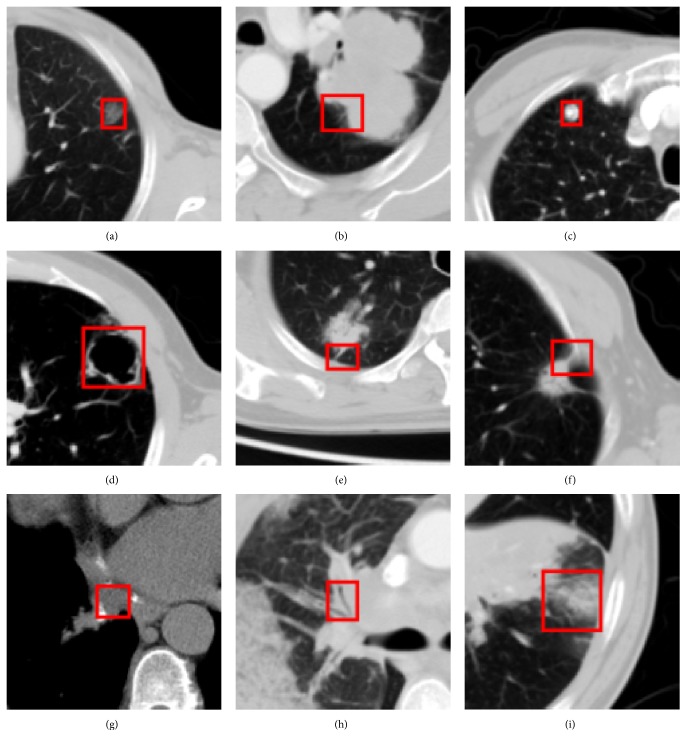
The examples of annotated CISLs in LISS database: (a) GGO, (b) lobulation, (c) calcification, (d) cavity and vacuoles, (e) spiculation, (f) pleural indentation, (g) bronchial mucus plugs, (h) air bronchogram, (i) obstructive pneumonia.

In the following experiments, all the CT images containing lobulation sign come from our LISS database [2], which can be downloaded from the website: http://www.iscbit.org/LISS.html. LISS database is a publicly available database of Lung CT Imaging Signs, and it contains 271 CT scans, in which 677 abnormal regions corresponding to 9 categories of common CT imaging signs of lung diseases (CISLs) are detected and labeled by radiologists.  Figure 9 shows some examples. For lobulation signs, LISS database contains 41 lobulation samples.

In this paper, we select the top 25 lobulation samples as the positive training set (according to the order listed in the annotated file). The remaining 16 samples are used as the test set. In order to enlarge the training set, we transformed the 25 training samples to obtain 100 training samples finally. Actually, we adjusted the annotated bounding box slightly to obtain several different positive samples. As for the negative training set, it is composed of two parts: (1) the imaging signs of other categories in LISS database and (2) other nonlobulation regions which are annotated manually using bounding box, such as the normal tissue and the CT image background. At last, 600 negative samples are collected in our experiments.

#### 3.1.2. Evaluation Criteria

For the evaluation of experimental results in this paper, we consider the following criteria.

(i) Average positive detection per image (APPI): the average number of positive detection outputs per CT image, which includes all the true positives (TP) and false positives (FP) for each image. From APPI, we can know how many positive detections are outputted in a quick glance. Because the unique output of detection method may be false, APPI should be used together with precision.
(ii) Sample number of true positive (SNTP): SNTP is the average number of true positive detections in the detection output for all images in the test set. A best detection technique would give more true positives.
(iii) Recall rate (also called sensitivity): the recall rate denotes the number of positive detections with respect to all elements of *T*, and it informs us of the chances of miss detection by analytical technique.
(iv) Precision rate: the precision rate denotes the number of positive detections with respect to all elements of true positive (TP) and false positive (FP), and it is defined as
  (4) Precision rate=true positive nums of detectionstotal nums of detections∗100%.
  The precision rate demonstrates how many true positives among all the detection results are, so it also tells us indirectly how likely the detection method produces a false alarm.
(v) *F*1 score: The *F*1 score is an integrated value of recall and precision as follows:
  (5) $$\begin{array}{c} F1=\frac{2\times P\times R}{P+R}, \end{array}$$
where *P*, *R* denote precision and recall, respectively.
(vi) Average time (AT): it is the average time consumed for processing each CT image.

#### 3.1.3. Parameter Setting

As mentioned above, we detected the lobulation by sliding window based methods. For the sliding window, because the smaller window cannot wrap the lobulation region annotated by radiologists and larger window will include superfluous information, we set window size to the mean size of bounding box annotated by radiologists (30 pixels) for all the other methods except the LIOP related method. Since the LIOP algorithm requires an odd value of window size, the window size of LIOP related methods is set to 31 in this paper.

In order to observe the influence of step size between two adjacent sliding windows, we repeated the evaluation on LISS database with step size set to 4, 6, 8, 10, 12, 14, 16, 18, and 20.

For the SVM classifier, the polynomial kernel function of 3 order and Sequential Minimal Optimization (SMO) are used in our experiments.

### 3.2. Experimental Results

#### 3.2.1. Effectiveness

In this section, we would find the optimal parameters and configuration of our algorithms and present the results from all the considered lobulation detection solutions.

Firstly, we performed A1 algorithm to test the effectiveness of a template matching method. For local feature based algorithms, the suitable encoding method was firstly decided through A2, A3, and A4 algorithms. Then the suitable features or mixture features were decided through the experiments based on A5–A9 algorithms. Finally, we performed A10a and A10b, respectively, to further reduce the false positive regions.

**Figure 10:**
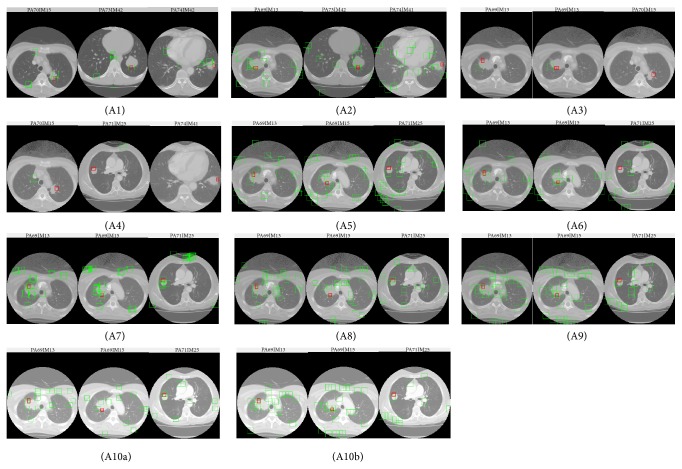
Example results of our experiments, where the red box surrounds the true lobulation sign (ground truth), and the green boxes are output of our solution.

Figure 10 illustrated the best result of each algorithm designed in this paper. Figure 10(A1) shows the result of template matching with step size 1, Figures 10(A2)–10(A9) show the results of Algorithms 2 to 9 with step size 14, 4, 4, 12, 16, 4, 18, and 18, respectively, and Figures 10(A10a) and 10(A10b) show the results of Algorithms 10a and 10b with step size 18. Each algorithm achieves its best result with specific step size.

The detailed detection results of our designed algorithms were shown in Table 2, in which step size means the step size of the sliding window. Through the results in Table 2, we obtain the following experimental discoveries.

1. Template matching method (A1) is very sensitive to sliding step size. When step 4 or longer one is used, the algorithm cannot detect any positive samples.
2. As described in the experiments of A2, A3, and A4 algorithms, for lobulation detection work, the BOF encoding method is the best one compared with VLAD and FV method. For all these three encoding methods, the step length of sliding window has a great influence on the detection results, especially from the view of APPI and AT criterion.
3. Comparing with Algorithm 2 (PHOW feature), Algorithm 5 (PHOW-HOG feature) reduced not only the APPI, but also the SNTP output slightly. The possible reason is the shape description ability of HOG feature. We also found that A6 (PHOW-SC feature) does not increase the recall rate of lobulation detection, while it has higher computing efficiency than HOG feature.
4. Algorithm 7 (GC feature) missed many positive ROIs, while the rate of false positive of A7 is the lowest one. On the other hand, the computing efficiency of GC feature is very low. Compared with global context feature, the shape context feature has better performance and computing efficiency for the lobulation detection task.
5. For the bending energy method (A10), it performed better than LIOP solution (A8) and LIOP_LBP solution (A9). The APPI is reduced by using the bending energy method, though the AT is increasing slightly (see Table 3). Among A8, A9, and A10, A10b achieves the best result with step 18. At the same time, the number of true positives detected in this solution is reduced slightly. In A10b, the outputs of LIOP_LBP feature are taken as the input of bending energy method.
6. GC feature (A7) stands out among all the features considered in this paper, achieving 0.1009 *F*1 score.

#### 3.2.2. Computational Efficiency

We further tested the efficiency of our presented algorithms. Note that all the experiments were performed on a computer with 3.2 GHz CPU and 10 GB memory. The computational efficiencies of our designed algorithms are illustrated in Table 3. We can find that the computational efficiency of template matching method is the highest one (with minimum time, about 7.5 s for all step sizes). Algorithm 10b brought the best *F*1 score measurement (for step size 18) and its total computation time is about 15.8 s (step size 18).

## 4. Discussions

We have designed several sliding window based algorithms to detect the lobulation signs in lung CT images. Further, we consider the bending energy to detect lobulation signs in this paper. To our knowledge, this is the first application of bending energy to lobulation detection. However, the current experimental results are still unsatisfactory for practical applications.

Through analyzing the experimental results carefully, we find that there are several challenges in detecting lobulation signs which are discussed as follows.

**Figure 11:**
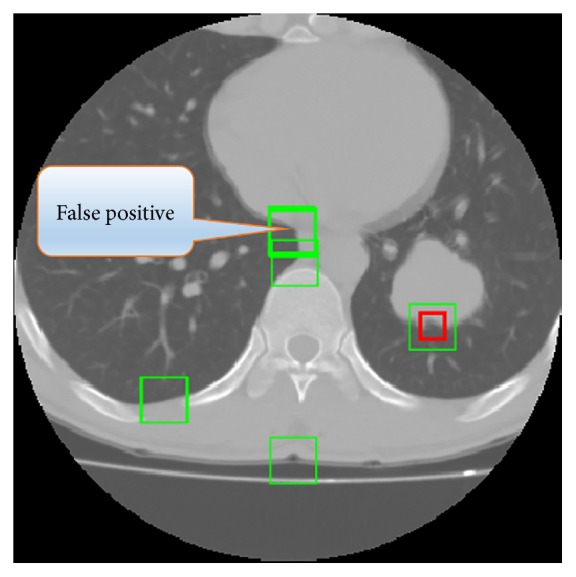
False positive analysis.

1. The shape description ability of used features is crucial for lobulation detection. The global context shape descriptor has impressive ability to detect lobulation signs alone, and the bending energy method has superior ability to reduce false positives.
2. Sliding window based methods suffer from locality of extracted features. The global context has better performance than shape context implying that global information is indispensable. In addition, as illustrated in Table 2, the step size of window based method is important factor affecting the detection result.
3. More global segmentation is not robust. Although the global context feature stands out (for *F*1 score criterion) among all the features considered in this paper, it also missed many positive ROIs.
4. It is challenging to detect lobulation signs relying on only its appearance information (shape and texture). For example, in Figure 11, we cannot assert that the region pointed by the arrow is not a lobulation sign by only appearance information. So if we expect excellent detection result, we must use more information, just like the radiologists do. For example, we may use the domain knowledge of tissue distribution to reduce the false positive regions (such as segmenting lung parenchyma more accurately or removing the airways in lung parenchyma in advance).

According to the challenges analyzed above, the possible strategies which can bring us closer to excellent detection result may include the following ones.

1. We may benefit from a better segmentation algorithm of lung parenchyma. In the above experiments, some false positive regions come from the area out of the lung parenchyma area.
2. We may use the important anatomic information, such as lung lobe distribution information. On the other hand, we may also utilize statistical distribution information of lobulation signs to improve the detection result. However, this method will need lots of positive samples.

## 5. Conclusions

So far as we know, there is no research work focusing on the automatic detection of lobulation imaging sign currently. The main contributions of this paper are summarized as follows. (1) We designed a sliding window based algorithm framework for lobulation detection from 2D lung CT images. Under the designed framework, three categories of automatic detection algorithms are implemented, including template matching, local feature based classifier, and bending energy. We evaluated the proposed methods through experiments and investigated the effects of these techniques. (2) We applied the bending energy to detect lobulation signs in this paper. To our knowledge, this is the first kind of work for direct lobulation detection and first application of bending energy to any kind of lobulation work.

Experimental results showed that the combined algorithm (A10a) of LIOP feature and BOF encoding method in local feature based classifiers has an optimal recall rate (100%), while the combination of GC feature and BOF encoding method (A7) has the best *F*1 score (0.1009) in all designed algorithms. Furthermore, the bending energy method is shown to be appropriate for reducing the false positives. The average positive detection per image was reduced from 30 to 22, and *F*1 score was increased to 0.0643 from 0.0599 when the bending energy method is performed by taking the results of A9 as its input. Based on our experimental results, we further discuss the challenges of lobulation detection and point out the possible approaches to promote the lobulation detection. We believe that the global feature and shape description ability are very important; however future research based only on local features likely will fail and should be avoided. Furthermore, more information need to be considered in lobulation detection work if we want to achieve excellent performance.

In future work, we will focus on these challenges and the strategies which may reduce the output of false positives, especially how to segment the lung parenchyma with larger lesion area more accurately.

## Figures and Tables

**Algorithm 1:**
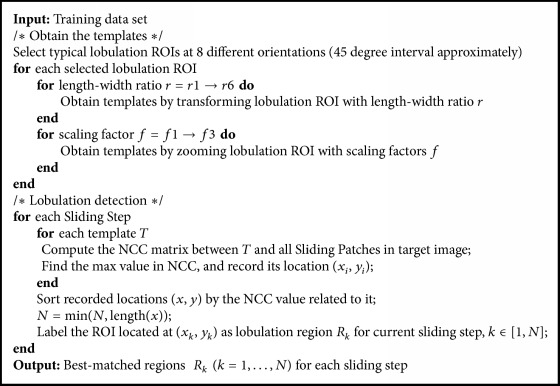
Template matching based lobulation detection.

**Algorithm 10:**
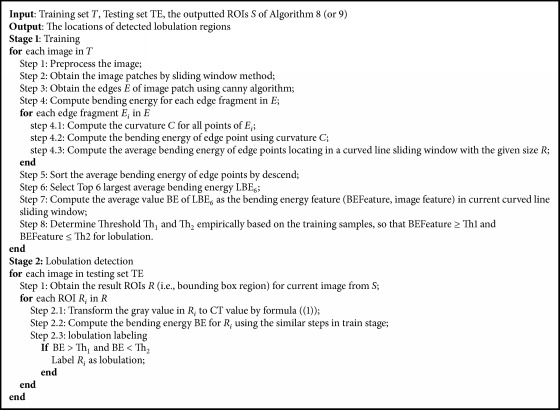
Bending energy based lobulation detection.

**Table 1:** Our lobulation detection algorithms based on local features.

Number	Features	Encoding methods
A2	PHOW	BOF
A3	PHOW	FV
A4	PHOW	VLAD
A5	PHOW-HOG	BOF
A6	PHOW-shape context	BOF
A7	PHOW-global context	BOF
A8	LIOP	BOF
A9	LIOP-LBP	BOF

**(a):** 

	Step size								
	1	2	4	6	8	10	12	14	16
A1									
SNTP	4	0	0	0	0	0	0	0	0
APPI	7.5	7.8	7.3	7.4	7.8	7.5	7.5	7.3	7.8
Recall	25%	0	0	0	0	0	0	0	0
Precision	2.92%	0	0	0	0	0	0	0	0
*F*1 score	0.0522								

**(b):** 

	Step size								
	4	6	8	10	12	14	16	18	20
A2									
SNTP	14	12	11	9	7	7	5	3	4
APPI	223.5	96.1	55	35.6	20.9	16.8	13.4	11.9	9.6
Recall	87.5%	75%	68.75%	56.25%	43.75%	43.75%	31.25%	18.75%	25%
Precision	2.43%	2.15%	2.5%	2.11%	2.69%	3.36%	2.79%	1.58%	3.25%
*F*1 score	0.0473	0.0417	0.0482	0.0407	0.0508	0.0624	0.0512	0.0291	0.0575

A3									
SNTP	0	0	0	0	0	0	0	0	0
APPI	0.2	0	0.1	0.1	0	0	0	0	0
Recall	0	0	0	0	0	0	0	0	0
Precision	0		0	0					
*F*1 score									

A4									
SNTP	0	0	0	0	0	0	0	0	0
APPI	1.9	0.8	0.6	0.1	0.1	0.3	0.2	0.1	0
Recall	0	0	0	0	0	0	0	0	0
Precision	0	0	0	0	0	0	0	0	
*F*1 score									

A5									
SNTP	14	9	10	8	6	4	3	0	2
APPI	190.2	84.9	49.4	27.4	19.8	15.8	12.9	10.5	6.1
Recall	87.5%	56.25%	62.5%	50%	37.5%	25%	18.75%	0	12.5%
Precision	2.37%	1.77%	2.41%	1.82%	2.85%	1.59%	1.45%	0	2.06%
*F*1 score	0.0461	0.0343	0.0463	0.0352	0.0529	0.0299	0.0269		0.0354

A6									
SNTP	12	10	9	9	4	5	6	2	2
APPI	198.6	83.6	49.1	30.6	18.9	16.9	12.25	11.1	7.8
Recall	75%	62.5%	56.25%	56.25%	25%	31.25%	37.5%	12.5%	12.5%
Precision	2.33%	2.39%	2.54%	2.66%	1.99%	1.85%	4.08%	1.13%	2.42%
*F*1 score	0.0452	0.0461	0.0487	0.0508	0.0368	0.0348	0.0736	0.0207	0.0405

A7									
SNTP	10	6	5	1	3	2	0	0	0
APPI	25.1	9.9	6.1	3.7	3.3	1.9	1.3	1.1	1.1
Recall	62.5%	37.5%	31.25%	6.25%	18.75%	12.5%	0	0	0
Precision	5.49%	5.03%	5.15%	1.69%	5.66%	6.67%	0	0	0
*F*1 score	**0.1009**	0.0887	0.0885	0.0267	0.0870	0.0870			

A8									
SNTP	16	16	15	10	10	7	7	7	5
APPI	564.5	257.8	140.3	87.4	66.3	46.2	37.8	28.8	21.1
Recall	100%	100%	93.75%	62.5%	62.5%	43.75%	43.75%	43.75%	31.25%
Precision	1.38%	1.38%	1.20%	1.0%	1.23%	1.22%	1.32%	1.52%	1.48%
*F*1 score	0.0273	0.0273	0.0238	0.0197	0.0241	0.0237	0.0257	0.0294	0.0283

A9									
SNTP	16	16	13	12	11	9	11	13	7
APPI	580.2	259.6	149.4	92.1	62.4	43.3	35.3	30.1	22.4
Recall	100%	100%	81.25%	75%	68.75%	56.25%	68.75%	81.25%	43.75%
Precision	2.09%	2.31%	2.0%	2.17	2.61%	2.16%	2.84%	3.11%	2.23%
*F*1 score	0.0409	0.0452	0.0392	0.0420	0.0502	0.0417	0.0545	**0.0599**	0.0424

A10a									
SNTP	16	16	14	8	10	7	5	6	4
APPI	368.5	170.1	91.1	56.1	45.3	28.1	24.1	19.3	13.9
Recall	100%	100%	87.5%	50%	62.5%	43.75%	31.25%	37.5%	25%
Precision	1.76%	1.84%	1.58%	1.0%	1.66%	1.78%	1.30%	1.94%	1.79%
*F*1 score	0.0347	0.0361	0.0310	0.0197	0.0322	0.0342	0.0249	0.0369	0.0335

A10b									
SNTP	15	16	13	12	11	9	10	11	5
APPI	447.8	200.1	114.4	70.1	48.1	35.1	26.9	22.3	16.8
Recall	93.75%	100%	81.25%	75%	68.75%	56.25%	62.5%	68.75%	31.25%
Precision	2.25%	2.59%	2.24%	2.32%	2.60%	2.50%	3.25%	3.37%	2.24%
*F*1 score	0.0439	0.0505	0.0436	0.0500	0.0501	0.0478	0.0618	**0.0643**	0.0418

**Table 3:** The consumed time (in seconds) of our presented algorithms.

Algorithm	Step size								
	4	6	8	10	12	14	16	18	20
A1	7.3	7.4	7.8	7.5	7.5	7.3	7.8	7.3	7.8
A2	233.9	104.3	58.9	35.6	27.6	19.9	15.6	12.0	10.5
A3	1539.1	687.8	390.2	251.3	176.1	127.7	100.3	76.1	65.2
A4	937.8	430.8	247.1	158.2	108.2	78.9	61.7	47.1	40.8
A5	318.3	142.5	80.9	52.4	36.5	26.7	20.9	16.0	13.6
A6	228.7	102.5	58.2	37.6	26.3	19.2	15.0	11.5	9.9
A7	748.6	335.6	190.2	122.9	86.0	62.7	49.2	37.3	32.1
A8	32.6	14.8	8.2	5.3	3.8	2.8	2.2	1.8	1.4
A9	227.9	101.4	57.6	37.2	26.1	19.0	14.9	11.3	9.7
A10a	101	45.7	25.3	16	11.9	8.3	6.8	5.3	3.9
A10b	313.1	139.3	79.5	50.9	35.3	25.3	20.1	15.8	13
